# A clinical prediction model for distant metastases of pediatric neuroblastoma: an analysis based on the SEER database

**DOI:** 10.3389/fped.2024.1417818

**Published:** 2024-09-19

**Authors:** Zhiwei Yan, Yumeng Wu, Yuehua Chen, Jian Xu, Xiubing Zhang, Qiyou Yin

**Affiliations:** ^1^ Department of Paediatric Surgery, Affiliated Hospital of Nantong University, Medical School of Nantong University, Nantong, China; ^2^ Cancer Research Center Nantong, Affiliated Tumor Hospital of Nantong University, Nantong, China; ^3^ Department of Pediatric Surgery, Affiliated Hospital of Nantong University, Nantong, China; ^4^ Department of Medical Oncology, Nantong Second Peoples Affiliated Hospital of Nantong University, Nantong, Jiangsu, China

**Keywords:** neuroblastoma, distant metastasis, SEER database, machine learning, predictive model

## Abstract

**Background:**

Patients with distant metastases from neuroblastoma (NB) usually have a poorer prognosis, and early diagnosis is essential to prevent distant metastases. The aim was to develop a machine-learning model for predicting the risk of distant metastasis in patients with neuroblastoma to aid clinical diagnosis and treatment decisions.

**Methods:**

We built a predictive model using data from the Surveillance, Epidemiology, and End Results (SEER) database from 2010 to 2018 on 1,542 patients with neuroblastoma. Seven machine-learning methods were employed to forecast the likelihood of neuroblastoma distant metastases. Univariate and multivariate logistic regression analyses were used to identify independent risk factors for building machine learning models. Secondly, the subject operating characteristic area under the curve (AUC), Precision-Recall (PR) curves, decision curve analysis (DCA), and calibration curves were used to assess model performance. To further explain the optimal model, the Shapley summation interpretation method (SHAP) was applied. Ultimately, the best model was used to create an online calculator that estimates the likelihood of neuroblastoma distant metastases.

**Results:**

The study included 1,542 patients with neuroblastoma, multifactorial logistic regression analysis showed that age, histology, tumor size, tumor grade, primary site, surgery, chemotherapy, and radiotherapy were independent risk factors for distant metastasis of neuroblastoma (*P *< 0.05). Logistic regression (LR) was found to be the optimal algorithm among the seven constructed, with the highest AUC values of 0.835 and 0.850 in the training and validation sets, respectively. Finally, we used the logistic regression model to build a network calculator for distant metastasis of neuroblastoma.

**Conclusion:**

The study developed and validated a machine learning model based on clinical and pathological information for predicting the risk of distant metastasis in patients with neuroblastoma, which may help physicians make clinical decisions.

## 1 Introduction

NB is an embryonic tumor of the autonomic nervous system originating in the neural crest tissue and can occur anywhere in the sympathetic nervous system (1), and is one of the most common malignant tumors in children, accounting for approximately 8% of childhood cancer cases and 15% of childhood cancer deaths (2). Nearly 70% of patients develop metastases after diagnosis, and the sites of metastasis include the abdomen, chest, neck, and pelvis (3, 4). Despite the latest immunotherapy and other treatment options, the 5-year survival rate of high-risk patients is less than 45% (5), and recurrence or deterioration at the metastatic site is the most important cause of death in patients with neuroblastoma (6). The International Neuroblastoma Staging System (INSS) is the most widely used staging system for neuroblastoma. However, the INSS is limited in its ability to stratify patients by risk prior to initiating treatment, as staging results are typically determined post-surgery or biopsy. Based on this, early evaluation of patients at high risk of distant metastasis is beneficial to improve the prognosis of patients with NB.

Machine learning is an important artificial intelligence component that is now being successfully applied to medicine (7). Machine learning enables the development of different predictive models based on clinical problems, thus predicting disease progression and improving prognosis before clinical symptoms appear (8). Compared to traditional statistical methods, machine learning algorithms are more flexible, computationally efficient, have high prediction accuracy, and minimize training errors (9, 10).

The Surveillance, Epidemiology, and End Results (SEER) database is a publicly available, representative cancer reporting system in the United States. The database is suitable for epidemiological studies of tumor incidence, prevalence, and treatments, as same as for in-depth studies of specific disease subgroups, such as neuroblastoma with distant metastases (11).

In this study, we utilized the SEER database to gather information on patients with neuroblastoma from 2010 to 2018, focusing on those with distant metastases. We constructed seven predictive models using machine learning algorithms, based on common clinicopathological factors. The performance of these models was evaluated using multiple metrics. Additionally, we explored factors influencing distant metastases in neuroblastoma. The best-performing model was then applied to clinical evaluation of patients at high risk for distant metastases. This approach aims to facilitate early diagnosis and improve prognosis for individuals with neuroblastoma.

## 2 Materials and methods

### 2.1 Study population

**Figure 1:**
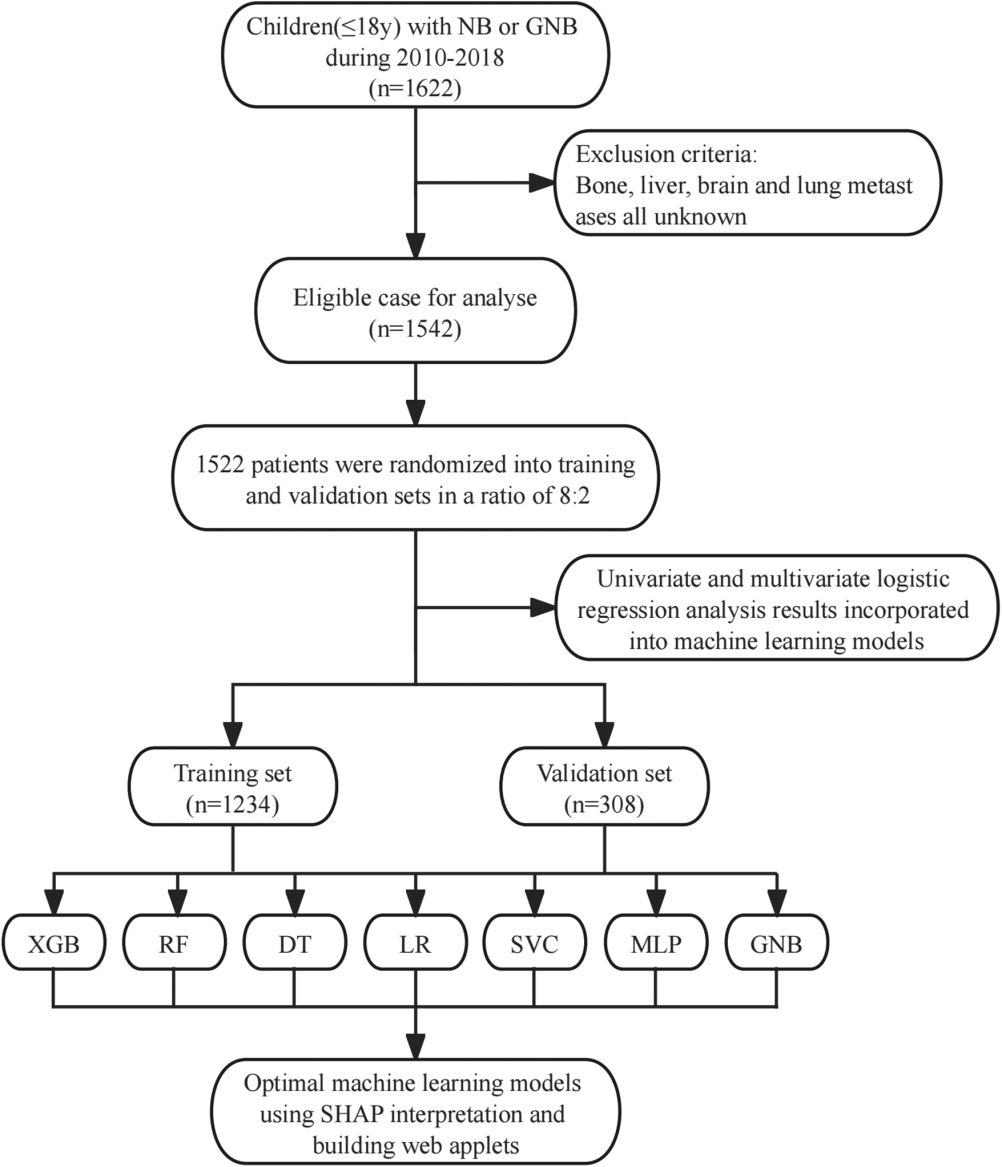
Study design flow chart. XGB, extreme gradient boosting; RF, random forest; DT, decision tree; LR, logistic regression; SVC, support vector machine; MLP, multilayer perceptron; GNB, Gaussian naive Bayes.

Data used in this study were obtained from the SEER Registry Research Database (www.SEER.concar.gov/seerstat). Based on the International Classification of Diseases for Oncology, Third Edition (ICD-O-3), researchers the case-listing section to select patients diagnosed with primary NB (ICD-O-3 histologic code: 9490, ganglioneuroblastoma; 9500, NB). The database did not start collecting information on lung, bone, liver, and brain metastasis sites until 2010. Therefore, we collected information on patients with NB diagnosed between 2010 and 2018 to analyze risk factors for distant metastasis. The clinical information of these patients included age, gender, race, histologic type, tumor size, tumor grade, primary site, laterality, presence of distant metastases, and history of surgery, chemotherapy, and radiation therapy. Exclusion criteria: cases older than 18 years and cases in which the presence of metastases was unknown and other information was unknown. The basic patient information in the SEER database can be found in Table 1, and the flowchart of the design of this study is shown in Figure 1.

**Table 1:** Clinical and pathological characteristics of the study population.

Variables	Training set	Validation set	*P*-value
*n*	1,234	308	
Age (years), *n* (%)			0.629
≤1	636 (41.2%)	154 (10%)	
>1	598 (38.8%)	154 (10%)	
Sex, *n* (%)			0.682
Male	649 (42.1%)	166 (10.8%)	
Female	585 (37.9%)	142 (9.2%)	
Race, *n* (%)			0.167
White	919 (59.6%)	219 (14.2%)	
Black	161 (10.4%)	53 (3.4%)	
Other	154 (10%)	36 (2.3%)	
Histology, *n* (%)			0.553
Neuroblastoma	1,016 (65.9%)	258 (16.7%)	
Ganglioneuroblastoma	218 (14.1%)	50 (3.2%)	
Tumor size (cm), *n* (%)			0.802
≤3	89 (5.8%)	23 (1.5%)	
3–6	221 (14.3%)	52 (3.4%)	
6–10	226 (14.7%)	57 (3.7%)	
≥10	302 (19.6%)	85 (5.5%)	
Unknown	396 (25.7%)	91 (5.9%)	
Tumor grade, *n* (%)			0.193
Grade I/II	24 (1.6%)	6 (0.4%)	
Grade III	554 (35.9%)	131 (8.5%)	
Grade IV	46 (3%)	20 (1.3%)	
Unknown	610 (39.6%)	151 (9.8%)	
Primary site, *n* (%)			0.211
Adrenal	632 (41%)	155 (10.1%)	
Retroperitoneum	126 (8.2%)	42 (2.7%)	
Other	476 (30.9%)	111 (7.2%)	
Laterality, *n* (%)			0.277
Unilateral	1,207 (78.3%)	298 (19.3%)	
Bilateral	27 (1.8%)	10 (0.6%)	
Bone metastases, *n* (%)			0.638
Yes	422 (27.4%)	111 (7.2%)	
No	811 (52.6%)	197 (12.8%)	
Unknown	1 (0.1%)	0 (0%)	
Brain metastases, *n* (%)			0.495
Yes	48 (3.1%)	11 (0.7%)	
No	1,178 (76.4%)	293 (19%)	
Unknown	8 (0.5%)	4 (0.3%)	
Liver metastases, *n* (%)			0.315
Yes	167 (10.8%)	41 (2.7%)	
No	1,063 (68.9%)	264 (17.1%)	
Unknown	4 (0.3%)	3 (0.2%)	
Lung metastases, *n* (%)			0.235
Yes	52 (3.4%)	17 (1.1%)	
No	1,175 (76.2%)	287 (18.6%)	
Unknown	7 (0.5%)	4 (0.3%)	
Surgery, *n* (%)			0.897
Yes	949 (61.5%)	234 (15.2%)	
No	279 (18.1%)	72 (4.7%)	
Unknown	6 (0.4%)	2 (0.1%)	
Chemotherapy, *n* (%)			0.717
Yes	828 (53.7%)	210 (13.6%)	
No/Unknown	406 (26.3%)	98 (6.4%)	
Radiotherapy, *n* (%)			0.633
Yes	325 (21.1%)	77 (5%)	
None/Unknown	909 (58.9%)	231 (15%)	

### 2.2 Modeling and evaluation

We use seven machine learning algorithms to construct the model including extreme gradient boosting (XGB) (12), random forest (RF) (13), decision tree (DT) (14), logistic regression (LR) (15), support vector machine(SVC) (16), multilayer perceptron (MLP) (17) and gaussian naive bayes(GNB) (18). Machine learning can be analyzed with large sample sizes, so we chose the SEER database, which can provide us with a large amount of sample data for model building. We randomly divided the samples into a training set and a validation set in the ratio of 8:2, trained seven models using the training set, and calculated the AUC values for each model separately. The AUC value is the area under the receiver operating characteristic curves (ROC) value, with values close to 1 indicating reliable predictive power and values close to 0.5 implying poor prognostic power (19). One of the major advantages of ROC analysis is that the performance of predictor variables can be estimated without a specific threshold, i.e., without a specific correlation with the threshold, making it more suitable for medical research (20). PR curves are also a graphical representation for evaluating the performance of machine learning models, and we use average precision to determine model accuracy. Additionally, to avoid false negatives or false positives in the results, we also used decision analysis curves (DCA) to further evaluate the clinical decision-making ability of the models, and calibration curves to evaluate the predictive ability of the models. In the validation set, we also calculated the AUC value of each model and used PR curves, decision curves, and calibration curves to further validate the reliability and predictive accuracy of the models. Finally, we select the model that performs best in both the training and validation sets as the optimal prediction model. Shapley's Additive Explanations (SHAP) is a method for interpreting predictions filtered by optimally integrated machine learning models (21). The interpretable model SHAP was used to assess the importance of each variable in the optimal model. Finally, a web calculator was created to facilitate the clinical use of the predictive model.

### 2.3 Statistical analysis

We used PyCharm (version 2023.3.3, www.JetBrains.com.cn) and Python (version 3.10, http://www.python.org) to statistically analyze and model clinicopathological information. Univariate and multivariate logistic regression analyses were used to identify clinicopathologic factors associated with distant metastasis of NB. Factors with *P *< 0.05 in univariate logistic regression analysis were analyzed by multivariate logistic regression. Factors with *P* < 0.05 in multivariate logistic regression analysis were finally identified as independent risk factors for distant metastasis of NB. These factors were incorporated into the construction of subsequent machine-learning models.

## 3 Results

### 3.1 Baseline population characteristics

This study included clinical and pathological information of 1,542 individuals with NB from the SEER database. The data were randomly divided into training and validation sets in a ratio of 8:2. Table 1 demonstrates the basic information of the study populations in the training and validation sets, as well as the objectivity of the randomized grouping (*P* > 0.05). In Table 2, we analyzed the differences between individuals with distant metastasis (DM+) and those without distant metastasis (DM−) among the 1,542 patients with NB. We included 11 clinicopathological factors: age, sex, race, histology, tumor size, tumor grade, primary site, laterality, surgery, chemotherapy, and radiotherapy. The analysis revealed that 901 individuals did not have distant metastases, while 641 individuals developed distant metastases. Compared to individuals without distant metastasis, those with distant metastasis were more likely to be older than 1 year, have neuroblastoma histology, tumor diameter greater than 10 cm, tumor grade III or IV, and primary site in the adrenal gland. They were also more likely to have undergone radiotherapy and chemotherapy, but less likely to have received surgical treatment. All these differences were statistically significant (*P* < 0.05). We found no significant differences between the groups with and without distant metastasis in terms of sex, race, and laterality (*P* > 0.05).

**Table 2:** Distant metastases and absence of distant metastases in the study population.

Variables	DM (−)	DM (+)	*P*-value
*n*	901	641	
Age (years), *n* (%)			<0.001
≤1	513 (33.3%)	277 (18%)	
>1	388 (25.2%)	364 (23.6%)	
Sex, *n* (%)			0.141
Male	462 (30%)	353 (22.9%)	
Female	439 (28.5%)	288 (18.7%)	
Race, *n* (%)			0.316
White	666 (43.2%)	472 (30.6%)	
Black	117 (7.6%)	97 (6.3%)	
Other	118 (7.7%)	72 (4.7%)	
Histology, *n* (%)			<0.001
Neuroblastoma	671 (43.5%)	603 (39.1%)	
Ganglioneuroblastoma	230 (14.9%)	38 (2.5%)	
Tumor size (cm), *n* (%)			<0.001
≤3	90 (5.8%)	22 (1.4%)	
3–6	175 (11.3%)	98 (6.4%)	
6–10	158 (10.2%)	125 (8.1%)	
≥10	193 (12.5%)	194 (12.6%)	
Unknown	285 (18.5%)	202 (13.1%)	
Tumor grade, *n* (%)			0.023
Grade I/II	22 (1.4%)	8 (0.5%)	
Grade III	385 (25%)	300 (19.5%)	
Grade IV	31 (2%)	35 (2.3%)	
Unknown	463 (30%)	298 (19.3%)	
Primary site, *n* (%)			<0.001
Adrenal	339 (22%)	448 (29.1%)	
Retroperitoneum	106 (6.9%)	62 (4%)	
Other	456 (29.6%)	131 (8.5%)	
Laterality, *n* (%)			0.222
Unilateral	883 (57.3%)	622 (40.3%)	
Bilateral	18 (1.2%)	19 (1.2%)	
Surgery, *n* (%)			0.006
Yes	717 (46.5%)	466 (30.2%)	
No	179 (11.6%)	172 (11.2%)	
Unknown	5 (0.3%)	3 (0.2%)	
Chemotherapy, *n* (%)			<0.001
Yes	426 (27.6%)	612 (39.7%)	
No/Unknown	475 (30.8%)	29 (1.9%)	
Radiotherapy, *n* (%)			<0.001
Yes	117 (7.6%)	285 (18.5%)	
None/Unknown	784 (50.8%)	356 (23.1%)	

DM (+), patients with distant metastasis; DM (−), patients without distant metastasis.

### 3.2 Univariate and multivariate logistic regression analysis

To identify risk factors associated with distant metastasis in NB, we performed univariate and multivariate logistic regression analyses. These analyses helped determine which variables should be included in our machine learning model. Univariate logistic regression analysis revealed that age, histology, tumor size, tumor grade, primary site, surgery, chemotherapy, and radiotherapy were significantly associated with distant metastasis in NB (*P* < 0.05, Table 3). Following this, we incorporated these factors into a multivariate logistic regression analysis. This analysis confirmed that all eight factors remained independent risk factors for distant metastasis in neuroblastoma (*P* < 0.05, Table 3). Eight independent risk factors were incorporated into the subsequent machine-learning model for the next step of model construction.

**Table 3:** Univariate and multivariate logistic regression analysis.

Variables	Total (*N*)	Univariate analysis		Multivariate analysis	
		Odds Ratio (95% CI)	*P*-value	Odds Ratio (95% CI)	*P*-value
Age (years)	1,542				
≤1	790	Reference		Reference	
>1	752	1.737 (1.416–2.132)	<0.001	1.987 (1.491–2.648)	<0.001
Sex	1,542				
Male	815	Reference			
Female	727	0.859 (0.701–1.052)	0.141		
Race	1,542				
White	1,138	Reference			
Black	214	1.170 (0.872–1.569)	0.295		
Other	190	0.861 (0.628–1.181)	0.353		
Histology	1,542				
Neuroblastoma	1,274	Reference		Reference	
Ganglioneuroblastoma	268	0.184 (0.128–0.264)	<0.001	0.425 (0.264–0.685)	<0.001
Tumor size (cm)	1,542				
≤3	112	Reference		Reference	
3–6	273	2.291 (1.352–3.883)	0.002	1.975 (1.019–3.829)	0.044
6–10	283	3.236 (1.921–5.454)	<0.001	1.636 (0.852–3.145)	0.139
≥10	387	4.112 (2.477–6.827)	<0.001	1.509 (0.803–2.833)	0.201
Unknown	487	2.900 (1.759–4.780)	<0.001	1.401 (0.750–2.617)	0.290
Tumor grade	1,542				
Grade I/II	30	Reference		Reference	
Grade III	685	2.143 (0.941–4.881)	0.070	0.486 (0.161–1.472)	0.202
Grade IV	66	3.105 (1.210–7.969)	0.018	0.278 (0.081–0.950)	0.041
Unknown	761	1.770 (0.778–4.028)	0.173	0.620 (0.204–1.880)	0.398
Primary site	1,542				
Adrenal	787	Reference		Reference	
Retroperitoneum	168	0.443 (0.314–0.624)	<0.001	0.398 (0.266–0.596)	<0.001
Other	587	0.217 (0.171–0.276)	<0.001	0.261 (0.194–0.351)	<0.001
Laterality	1,542				
Unilateral	1,505	Reference			
Bilateral	37	1.498 (0.780–2.878)	0.225		
Surgery	1,542				
Yes	1,183	Reference		Reference	
No	351	1.478 (1.163–1.879)	0.001	2.212 (1.600–3.057)	<0.001
Unknown	8	0.923 (0.220–3.881)	0.913	4.668 (0.870–25.034)	0.072
Chemotherapy	1,542				
Yes	1,038	Reference		Reference	
No/Unknown	504	0.042 (0.029–0.063)	<0.001	0.066 (0.042–0.102)	<0.001
Radiotherapy	1,542				
Yes	402	Reference		Reference	
None/Unknown	1,140	0.186 (0.145–0.239)	<0.001	0.468 (0.344–0.638)	<0.001

### 3.3 Construction and evaluation of machine learning models

**Figure 2:**
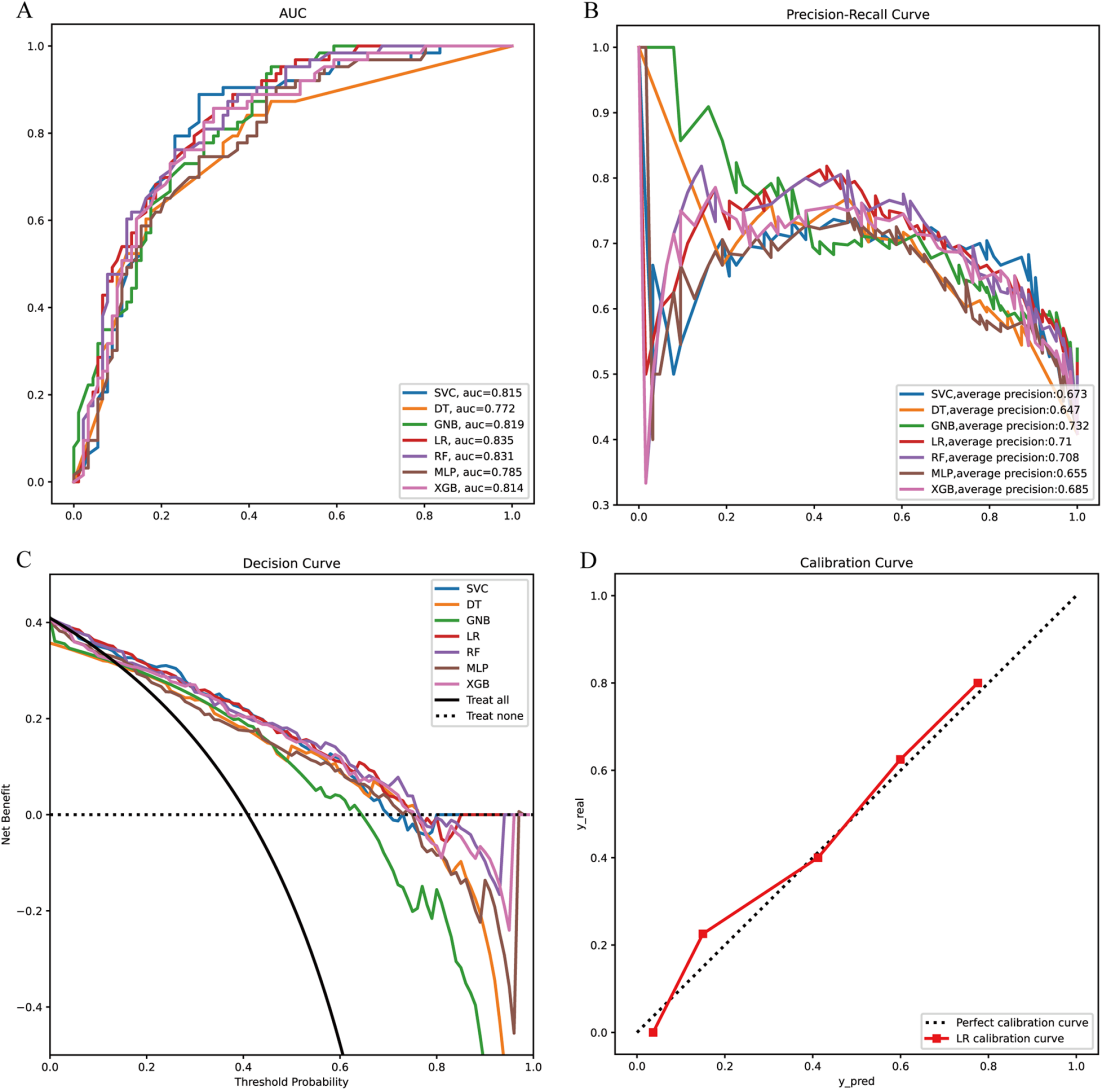
Model construction and evaluation. **(A)** ROC curves of 7 machine learning models in the training set. **(B)** PR curves of 7 machine learning models in the training set. **(C)** DCA curves of 7 machine learning models in the training set. **(D)** Calibration curves of the optimal models in the training set. XGB, extreme gradient boosting; RF, random forest; DT, decision tree; LR, logistic regression; SVC, support vector machine; MLP, multilayer perceptron; GNB, Gaussian naive Bayes.

We constructed prediction models using seven different machine learning algorithms in the training set. To verify the reliability of these models, we plotted the receiver operating characteristic (ROC) curves for each model and calculated the area under the curve (AUC), an index for evaluating prediction accuracy. All models demonstrated good prediction performance with AUC values greater than 0.70. The Logistic Regression (LR) model showed the highest AUC at 0.835 (Figure 2A). The precision-recall (PR) curves of the seven models in the training set revealed that the LR model had an average precision of 0.71, further confirming its reliable prediction performance (Figure 2B).

We also conducted a Decision Curve Analysis (DCA) with “Treat All” and “Treat None” as reference lines (Figure 2C). When the prediction curve lies above these two lines, it indicates that the model has a high prediction accuracy, which can be seen in Figure 2C where the LR model has a high prediction accuracy. Finally, we plotted the calibration curve of the LR model to assess the calibration of the prediction model (Figure 2D). The reference line through the origin serves as the baseline. A higher overlap between the calibration curve and this baseline indicates a smaller prediction error. As observed, the calibration curve of the LR model shows a high degree of overlap with the baseline, suggesting a smaller prediction error.

### 3.4 Model validation

**Figure 3:**
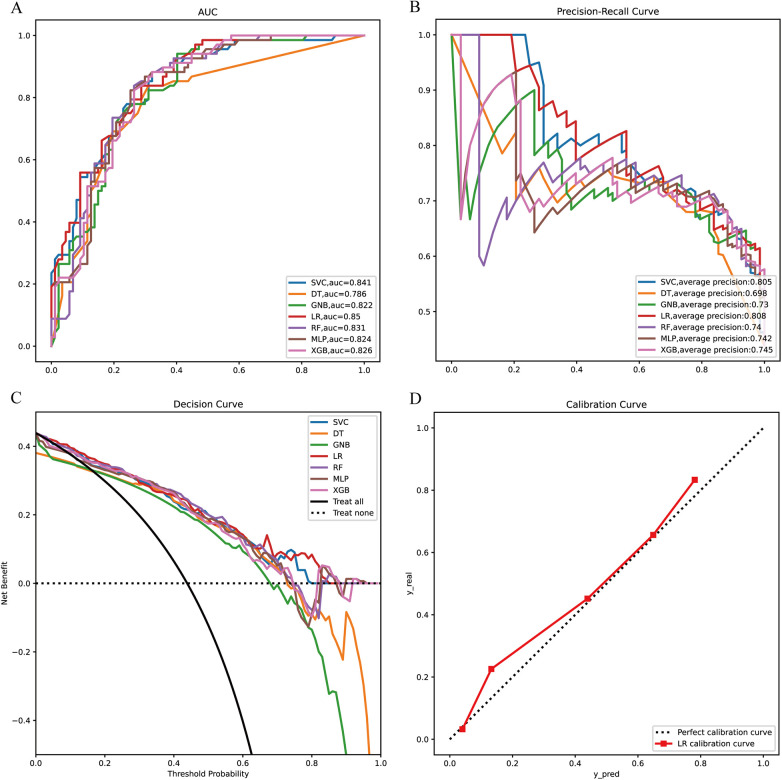
Model validation **(A)** ROC curves of the seven machine learning models in the validation set. **(B)** PR curves of the seven machine learning models in the validation set. **(C)** DCA curves of the seven machine learning models in the validation. **(D)** Calibration curves of the optimal models. XGB, extreme gradient boosting; RF, random forest; DT, decision tree; LR, logistic regression; SVC, support vector machine; MLP, multilayer perceptron; GNB, Gaussian naive Bayes.

**Figure 4:**
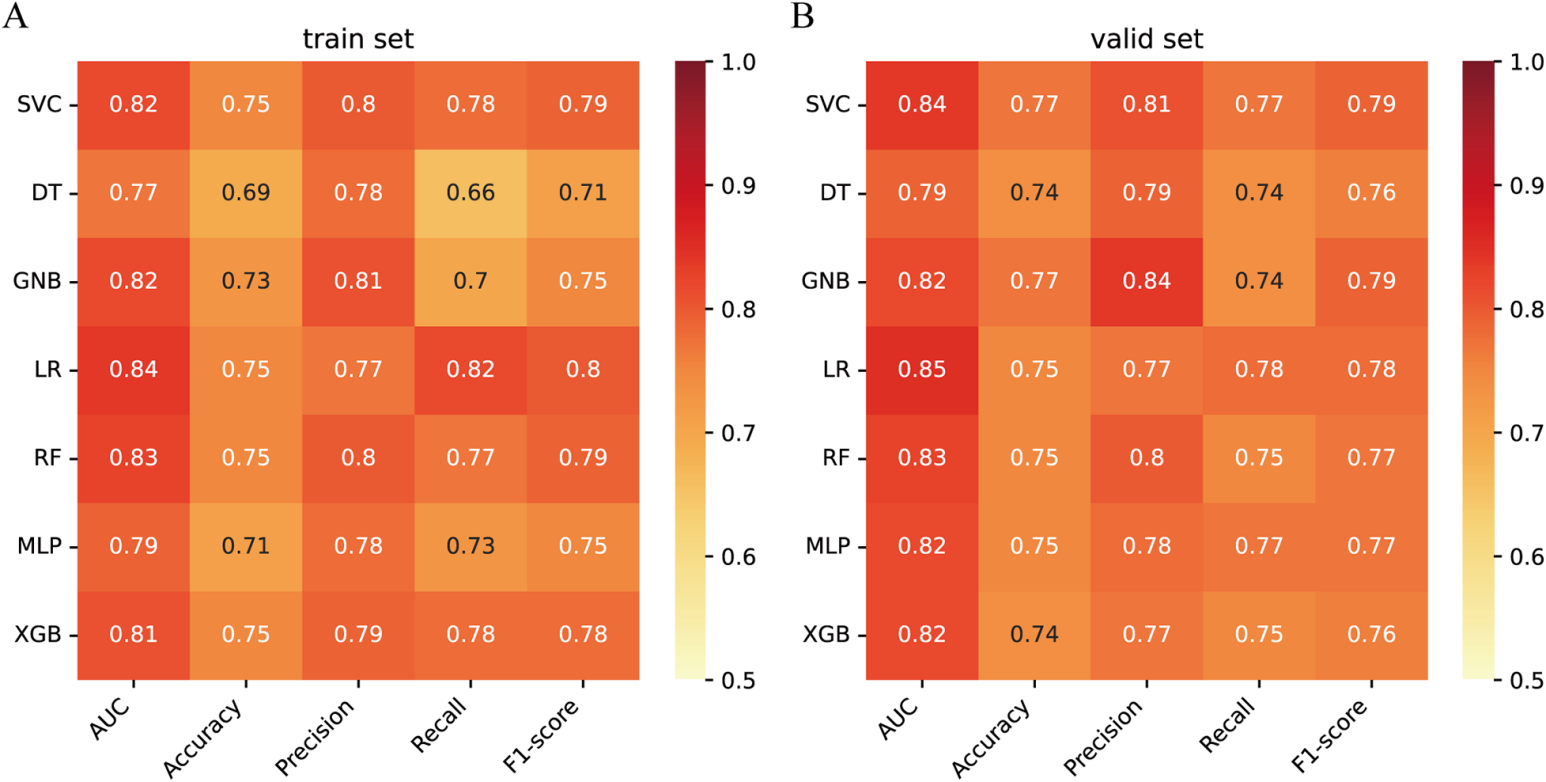
Performance prediction **(A)** heatmap of the performance of 7 machine learning models in the training set. **(B)** Heatmap of the performance of 7 machine learning models in the validation set.

Next, the seven machine learning models are validated using the validation set. Figure 3A shows the ROC curves of the seven machine learning models, and it can be seen that the LR model has the best prediction performance (AUC = 0.85). Figure 3B is the PR curve, and the LR model with AP = 0.808 also demonstrates very good prediction accuracy. In decision curve analysis, the LR decision curves all lie above both the Teat All and Teat None lines, demonstrating excellent prediction accuracy (Figure 3C). The calibration curve of the LR model also demonstrates the tiny prediction error, reflecting excellent prediction accuracy (Figure 3D). In addition, we use heatmaps to comprehensively show the performance of the seven machine-learning models from multiple dimensions (Figure 4). In summary, after developing and validating various machine learning models, the LR model emerged as the optimal predictor of distant metastasis in neuroblastoma, demonstrating the best performance and highest accuracy.

### 3.5 Explanation of optimal models for machine-learning

**Figure 5:**
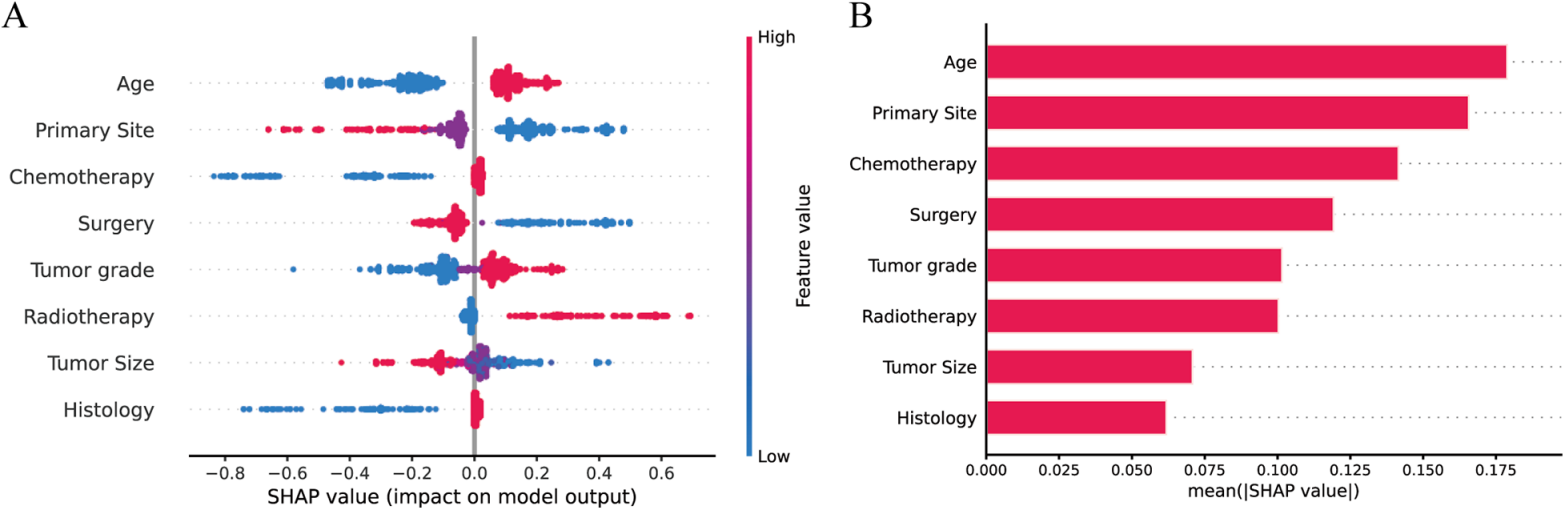
Explaining the relative importance of each variable in the LR model based on SHAP **(A)** Red represents a positive impact on the model; blue represents a negative impact on the model. **(B)** Visualization of results.

To visualize the machine learning algorithm, we use SHAP to interpret the relative importance of each variable in the LR model. In the SHAP analysis, the red color represents a positive impact on the model, while the blue color represents a negative impact on the model. The results revealed that age was the most important variable for predicting distant metastasis in neuroblastoma, followed by primary site, chemotherapy, surgery, tumor grade, radiotherapy, tumor size, and histology (Figure 5).

### 3.6 Web calculator

**Figure 6:**
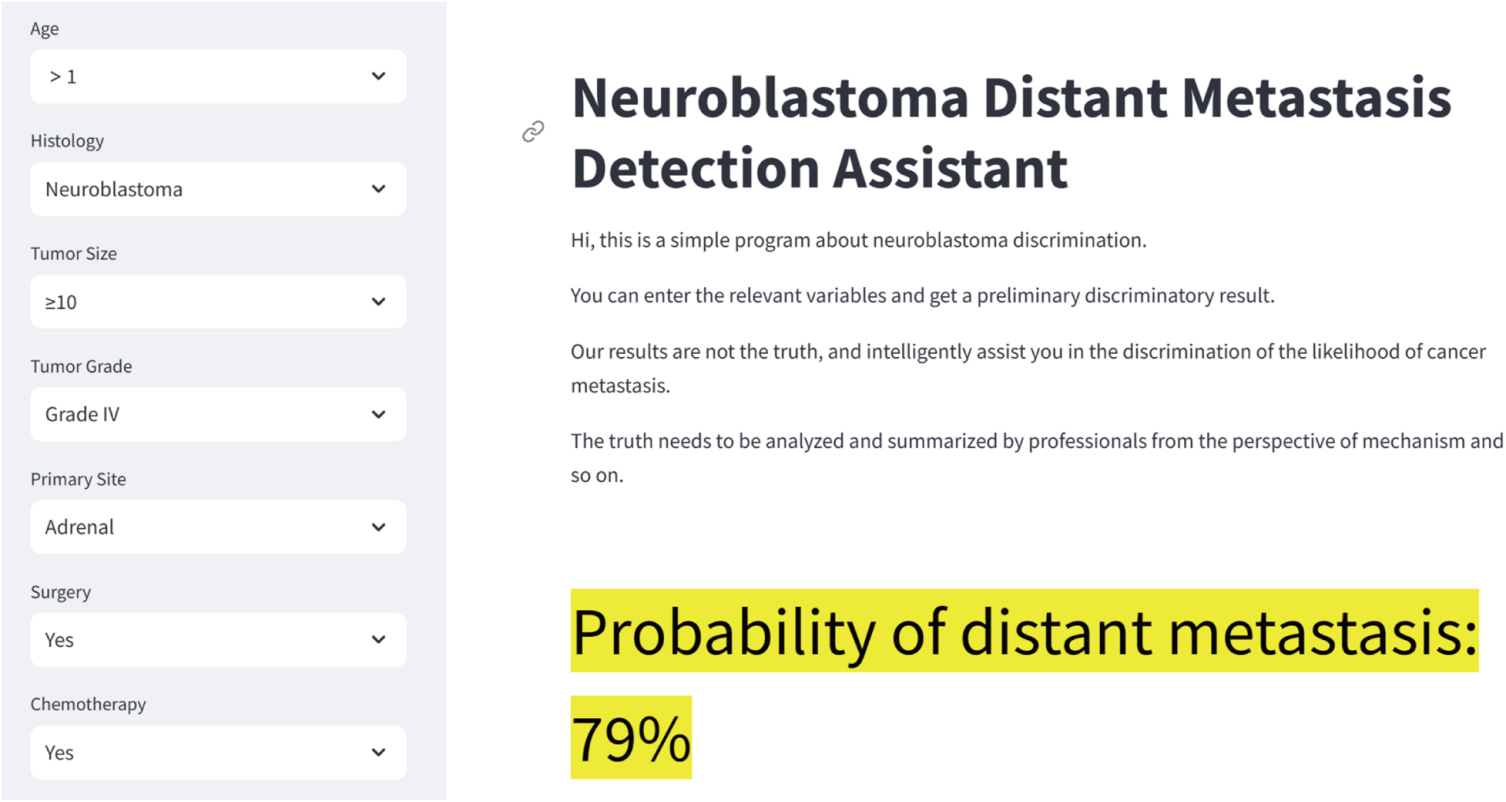
Neuroblastoma distant metastasis web calculator. The link to the web calculator is https://neuroblastoma-distant-metastasis-detection-assistant-bagytzysf.streamlit.app/.

After validation, the LR model has the best performance and accuracy among the seven machine learning prediction models. However, its computational complexity makes it challenging to use directly in clinical work. To address this, we developed a web-based calculator based on the LR model (Figure 6). This web-based calculator enables clinicians to quickly assess the probability of distant metastasis by inputting patient data, thereby facilitating the clinical application of our prediction model. The link to the web calculator is https://neuroblastoma-distant-metastasis-detection-assistant-bagytzysf.streamlit.app/.

## 4 Discussion

NB has been called the “king of childhood cancers”, and its tumor heterogeneity results in a 5-year survival rate of approximately 45% in children with high-risk NB (5), which may be strongly related to the lack of specific clinical manifestations and effective early detection methods. NB has an insidious onset, early symptoms are not obvious, and some children have distant metastases to bone and bone marrow at the time of diagnosis (22, 23). A recent study demonstrates that children with distant metastases have shorter overall survival times and worse prognoses compared to those without distant metastases. Furthermore, even in developed regions such as Europe and the United States, effective treatments are not readily available for many children with distant metastases (24, 25). The International Neuroblastoma Staging System (INSS) is the most widely used staging system for neuroblastoma. However, the INSS is limited in its ability to stratify patients by risk prior to initiating treatment, as staging results are typically determined post-surgery or biopsy. Our study addresses this limitation by constructing a neuroblastoma distant metastasis prediction model. This model utilizes relevant clinical information to accurately predict the likelihood of distant metastasis in patients. By facilitating early identification of high-risk patients, our model enables clinicians to make more timely diagnoses and develop appropriate treatment plans. Early intervention based on our predictive model can improve patient outcomes by allowing for prompt and targeted treatment strategies.

To the best of our knowledge, this study is the first to use a machine learning algorithm to construct a predictive model for distant metastasis of NB. In this study, we utilized compliant data from the SEER database to identify independent risk factors for distant metastasis in neuroblastoma We then used these factors to develop various machine learning models, with the LR model emerging as optimal after training and validation in independent sets. Logistic regression is an effective and powerful method for analyzing the impact of a set of independent variables on a binary outcome, which quantifies the unique contribution of each independent variable (26). Regression techniques are widely used in medical research because they can measure associations, predict outcomes, and control the effects of confounding variables, for example, logistic regression analysis played a very important role in prognostic studies of patients with renal cell carcinoma (27), and in studies of oral cancer incidence (28).

After screening the optimal model by machine learning, the effect of each variable in the model on distant metastasis of NB was evaluated using SHAP. As shown in Figure 5, all variables had an impact on distant metastasis of NB, with age taking the first place. Studies have shown that children with NB younger than 1 year of age have better overall and cancer-specific survival than other children (29), and children younger than 1 year of age are less likely to develop bone metastases compared to the rest of the children (24). We conjecture that this may be related to spontaneous tumor regression, which is prevalent in patients with NB younger than 12 months of age (30), and that spontaneous tumor regression may be associated with (I) neurotrophin deprivation, (II) loss of telomerase activity, (III) humoral or cellular immunity and (IV) alterations in epigenetic regulation and possibly other mechanisms (31).

Secondly, the primary site of the tumor was the second most important factor after age. Our study found that children whose primary site was located in the adrenal gland were more likely to develop distant metastases, which is consistent with the results of other studies (32). This may be because primary adrenal NB is more likely to have aberrant DNA structure due to amplification of the MYCN gene, as well as alterations in the tumor microenvironment (33, 34), resulting in tumors that are more likely to develop distant metastases. Tumor size is a risk factor for distant metastasis, but there are no clear conclusions showing that exactly how large a tumor is is positively associated with metastasis. Some studies have shown that NB with a diameter greater than 10 cm is more likely to metastasize (24), which is consistent with our study, and others have shown that the optimal tumor size threshold for overall survival in patients with NB is 4 cm (35). Also, surgery is an associated factor for NB metastasis, and our study showed that children who underwent surgery were less likely to develop distant metastases than those who did not. For neuroblastoma, surgical resection is an indispensable step in the whole treatment process (36). The goal of 90% tumor resection is the best approach at this stage, and the tumor should be removed as much as possible for the child, so that distant metastasis of the tumor can be avoided, and the prognosis can only be improved (37). As the most important adjuvant treatments for NB, chemotherapy, and radiotherapy also play an indispensable role in the metastasis of neuroblastoma. On the one hand, chemotherapy can reduce the size of the tumor and create conditions for surgery; on the other hand, some scholars have found that chemotherapy may increase the metastasis of malignant tumors, which is due to the fact that chemotherapy promotes the expression of metastatic genes and the secretion of metastatic exosomes (38).

Finally, the pathological grade of the tumor is also a risk factor for metastasis and the less differentiated the tumor (grade III/IV) the more likely it is to develop distant metastasis, this is possibly related to (I) uncontrolled cell proliferation and differentiation, (II) increased angiogenesis, (III) decreased intercellular adhesion, and (IV) immune escape, avoiding the immune system (39). Our study adequately incorporated some clinical as well as pathological factors of NB and the constructed machine learning model demonstrated good predictive performance. Finally, we constructed a NB distant metastasis web page calculator that can be better generalized for clinical use.

Despite the groundbreaking nature of our study, there are some limitations of this study. First, due to the fact that some variables were missing from the SEER database, we removed the missing data, which may have biased the results. Second, the study lacked further validation from external data. Finally, the lack of important information in the SEER database, such as chemotherapy regimen, immunotherapy, targeted therapy, stem cell transplantation, INSS staging, and whether MYCN was amplified, limited our further optimization of the model, and we will further improve it in the future by incorporating a variety of other clinical factors to better assist clinicians.

## 5 Conclusions

In conclusion, we constructed a prediction model for the risk of distant metastasis in patients with NB using machine learning algorithms. The LR model was found to have optimal predictive ability, showing high sensitivity, specificity, and accuracy with strong discriminative ability on both test and validation sets. We hope that this prediction model can help clinicians screen patients at high risk for distant metastasis of NB, intervene early to prevent distant metastasis of NB and improve patient prognosis.

## Data Availability

The original contributions presented in the study are included in the article/Supplementary Material, further inquiries can be directed to the corresponding authors.
